# Video feedback combined with peer role-playing: a method to improve the teaching effect of medical undergraduates

**DOI:** 10.1186/s12909-024-05040-x

**Published:** 2024-01-19

**Authors:** Jiwu Wang, Birong Wang, Dan Liu, Yiqun Zhou, Xin Xing, Xianggui Wang, Wei Gao

**Affiliations:** 1 Department of Cardiothoracic Surgery, Tianyou Hospital Affiliated to Wuhan University of Science and Technology, Wuhan, 430061 Hubei China; 2 Department of Cardiothoracic Surgery, Puai Hospital Affiliated to Jianghan University/Wuhan Fourth Hospital, Wuhan, 430030 Hubei China; 3 Department of thyroid gland and breast Surgery, Puai Hospital Affiliated to Jianghan University/Wuhan Fourth Hospital, Wuhan, 430030 Hubei China; 4 Department of Operating Room, Tongji Hospital, Tongji Medical College, Huazhong University of Science and Technology, Wuhan, 430030 Hubei China; 5 Department of Science Research and Education, Puai Hospital Affiliated to Jianghan University/Wuhan Fourth Hospital, Wuhan, 430030 Hubei China

**Keywords:** Video feedback, Peer role-playing, Problem-based learning, Teaching mode, Implementation effect

## Abstract

**Objective:**

The purpose of this study was to investigate the effectiveness of implementation of video feedback combined with peer role-playing (PRP) teaching method in medical undergraduates adopting problem-based learning (PBL) teaching mode.

**Methods:**

The undergraduates of five-year clinical medicine who get enrollment of Wuhan local University from 2016 and 2018 were selected to be the research objects. The same grade level is randomly divided into several groups to carry out PBL, with 6–10 students in each group. Following the principle of voluntary participation, 34 students were enrolled in the study group and 33 students in the control group finally. The research regards group as the unit, and study report in group should be carried out to fulfill the research. In the study group, the students were asked to perform PRP report, and the report videos were used for feedback. At the same time, the control group reported by PPT, and the feedback was carried out according to the PPT. At the end of the study, the “Competency Improvement Satisfaction Questionnaire (CISQ)” was distributed to investigate students’ satisfaction with this teaching method to improve their ability, Arizona Clinical Interview Score (ACIR) was administered in Chinese by a trained teacher unrelated using PRP method to assess students’ clinical inquiry ability and communication skills, and theory test was performed to assess mastery of theoretical knowledge.

**Results:**

The results show that the study group is superior to the control group in improving the interest of learning and the ability of independent learning, interpersonal communication and active problem solving. Although it is in terms of the confidence in becoming a real doctor and the ability of teamwork, language expression, clinical thinking cultivated, active knowledge acquired and understood that study group are better than the control group, the difference was not statistically significant. ACIR shows that the study group is significantly better than the control group in organization, timeline planning, and transition statements, openly questioning, smooth progress, and avoiding repetition, summarizing, understandable language, documentation and total score. There is no significant difference in eye contact and no interruption. The differences between the two groups are not statistically significant in terms of responsing to concerns, positive feedback, and additional questions. The theoretical test scores of the study group are significantly higher than those of the control group.

**Conclusion:**

Video feedback combined with peer role-playing teaching method implemented in medical undergraduates adopting PBL teaching mode is effective, it could stimulate interest in learning actively, improve interpersonal communication ability, improve learning efficiency and clinical knowledge and skills, and improve the confidence of becoming a real doctor. It is worthy of further research and promotion.

## Introduction

In the 1950s, Case Western Reserve University took the lead in carrying out the organ-Systems-based curriculum (OSBC) reform, and at the same time, some medical colleges in China also began the practice of OSBC teaching reform [1]. In 1969, McMaster University in Canada established problem-based learning method (PBL) [2] to reverse the situation that graduates who were trained in a traditional way were not competent to solve clinical problems. Compared with traditional lecture-based learning, PBL can increase students’ learning enthusiasm, cultivate students’ critical thinking, promote the integration of knowledge, improve students’ clinical thoughts and skills, and enhance the quality of medical teaching [3–5]. Since then, the teaching reform practice of using PBL in OSBC has been carried out all over the world. In 2015, in order to adapt to the teaching reform of OSBC and PBL, China published a series of textbooks on organ system integration planning for medical undergraduates, and more and more universities are using this textbook for teaching in China.

In a pedagogical context, feedback refers to information that informs learners about the actual state or performance of learning in order to regulate further learning processes in the direction of the learning standard pursued. Feedback is an essential component of the learning process, it is often lacking due to competing priorities. Feedback-lacking is a recurring theme when talking to the topic of medical education, which hinders the learning of clinical skills and leads to decreased interest and satisfaction of medical students. By contrast, high-quality feedback and coaching will promote learning and growth as well as personal and professional development [6].

Feedback can be provided by external sources or generated internally by learners. External feedback is provided and generated by sources (e.g., teachers, peers, and parents) other than the person receiving the feedback. External feedback can influence performance and motivation, but there is instability to this effect. Expert feedback (EF) is the most common type of external feedback, but its implementation remains challenging due to busy schedules, teaching workload, and large student population in medical education [7]. In contrast, peer feedback (PF), another form of external feedback, is increasingly being explored as a potential solution due to growing evidence supporting its effectiveness in increasing knowledge and skills, and improving patient safety culture [8]. It difficult to achieve reliability in PF as the feedback quality is highly dependent on their competency level and experience, and is affected by multiple factors [9]. If not well managed, learners could be misled. Feedback may be substandard due to memory limitations or missed observations, which can be overcome using techniques such as video recording [10]. Video feedback (VF) was an informative tool for skills refinement and a powerful means of richly supporting student learning [11]. Peer video feedback (PVF) allows students to participate more in learning and practice, which helps to improve students’ technical, efficiency and communication skills [12, 13]. A systematic review of has shown that PVF can significantly improve technical skills, but the ambivalent results have emerged for non-technical aspects [9].

Research shows that for learning to be most effective, students need to be active, and that a simulated clinical environment may provide benefits [14]. Peer role-play (PRP) is a training method based on simulation-based training method (SBTM), which has become the core educational strategy of professional skills-learning in the field of healthcare in the past 20 years [15],. in which medical students take turns to play the role of the patient and the clinician respectively. To rehearse the play is not the only purpose to perform this kind of teaching activity. What more important is to simulate the situation in clinical practice which we have to learn to cope with. Additionally, it is also one of the main purposes that to combine theory with practice which helps to prompt the enthusiasm of learning and the ability of comprehensive analysis and communication ability [16]. Thanks to the dependence of SBTM on entertaining and proactive methods [17], PRP has a good level of acceptability among students [18].

Internal feedback is generated by learners through inevitable spontaneous comparisons with prior standards or with their surrounding peers [19]. A central goal of feedback is to help reduce the gap between the current state of learning and the desired state of learning. Yet, achieving this goal requires a complex interplay of internal and external feedback.

## Objective

At present, there are relatively few studies on the combination of video feedback and peer role-playing in medical education. The purpose of this study was to investigate the effectiveness of implementation of VF combined with PRP teaching method in medical undergraduates adopting PBL teaching mode.

## Participants

The five-year clinical medicine undergraduates of Jianghan University in grade 2016 and 2018 were selected as the research objects. All clinical courses are taught in accordance with the organ-systems integration textbook. The same grade levels are randomly divided into several groups to carry out PBL, with 6–10 students in each group. Following the principle of voluntary participation, 34 students were enrolled in the study group and 33 students in the control group finally.

## Research methods

Preparing for PBL course after the first class, in study group, two students in one group were asked to search for information by themselves and wrote short scripts about the process of consultation and physical examination between doctors and patients according to the content they discussed and the information they searched. Students should rehearse by themselves before class and sent the short script to the instructor as an assignment before the second class. In the second class, each group performed this short role play according to the script for the first time, while the instructor made a live video. After performing, the video was feed back to the students who were demanded to watch the videos of all groups. When a video was played, the evaluation, analysis and feedback were asked to be given by their own group and others respectively. When completed, the instructor summarized and made the comments, pointing out the advantages and disadvantages. In the same way, students wrote a small play again after the second act as required. In the third class, they first conduct peer role play report, but the roles must exchange, and then conduct video feedback.

**Fig. 1:**
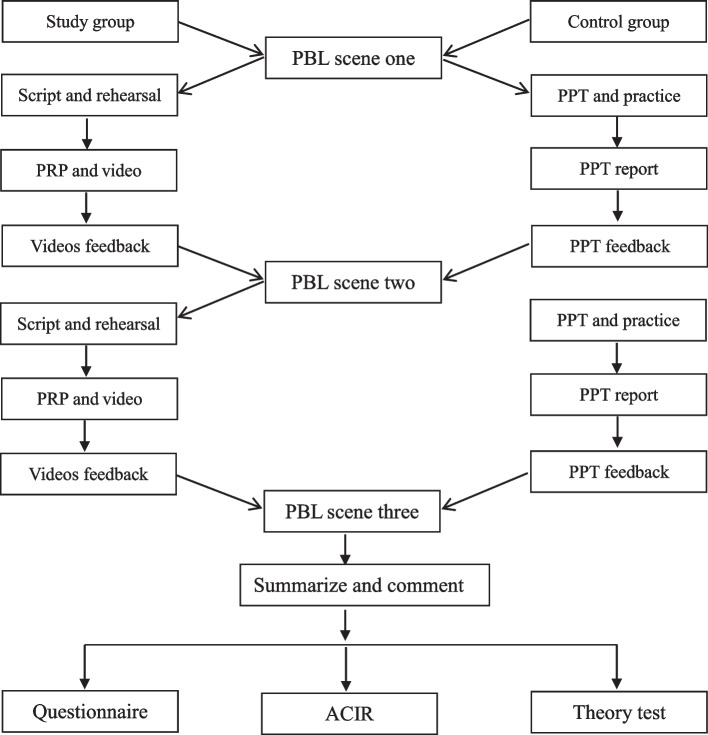
An overview of the study design

By contrast in the control group, after the first act, two students in one group were asked to search for information by themselves and made PPT then sent PPT as homework to the instructor by email before the second class. In the second class, PPT presentation will be conducted by groups firstly. After each group reported, the PPT was shown to students one by one. Each student had to see the PPT of his group and the other groups. The same as the study group, after presentation the evaluation analysis and feedback are made by all groups. After the completion of all PPT feedback, the instructor gave the summaries and comments, pointing out the positive and the negative. In the same way, make PPT as required after the second act. In the third class, make PPT report first and then give feedback. The design scheme is shown in Fig. 1.

The PBL course is divided into three scenes. In the study group, the students were asked to perform peer role play report, and the report videos were used for feedback. At the same time, the control group reported by PPT, and the feedback was carried out according to the PPT. The “ Competency Improvement Satisfaction Questionnaire (CISQ)”, Arizona Clinical Interview Score (ACIR) and theory test, were performed for both groups at the end of the study.

### Evaluation of performance

At the end of the course, the self-developed “Competency Improvement Satisfaction Questionnaire (CISQ)” was distributed to investigate students’ satisfaction with this teaching method to improve their ability. The contents of the questionnaire include ten aspects: improving learning interest, independent learning ability, teamwork ability, clinical thinking ability, interpersonal communication ability, active problem solving ability, active knowledge acquisition ability, and language expression ability, understanding knowledge points in the course, and confidence to become a real doctor. Options include yes, neutral, and no.

The Arizona Clinical Interview Rating (ACIR) [20] was administered to all students in Chinese by a trained teacher unrelated using PRP method. ACIR includes 14 items, with a maximum of 5 points for each item, and 70 points in total. This includes organization, timeline planning, transition statements, openly questioning, smooth progress, avoiding repetition, summarizing, understandable language, documentation, eye contact, no interruption, responsing to concerns, positive feedback, and additional questions. Compare the scores of each item and total scores between the two groups.

The test questions were randomly selected from the question bank of Jianghan University for theoretical assessment, and then the scores of the two groups were compared.

## Data analysis

SPSS17.0 software was used for statistical analysis. Chi-square test was used for counting data and T test was used for measurement data. *P* < 0.05 was considered statistically significant.

The two groups were aged between 20 to 22 years old. 34 undergraduates were enrolled in the study group, including 17 boys (50.0%) and 17 girls (50.0%), while 33 undergraduates were enrolled in the control group, with 14 boys (42.4%) and 19 girls (57.6%). There were no statistically significant difference between two groups (*P* = 0.627).

The results of the CISQ are shown in Table 1. Survey shows that the study team is better than the control group in all ten aspects. Among them, the study group is significantly excellent in improving learning interest, autonomous learning ability, interpersonal communication ability and active problem solving ability, the difference is statistically significant (*P* < 0.05). Although the study group is better than the control group in improving teamwork ability, clinical thinking ability, active knowledge acquisition ability, language expression ability, understanding knowledge points in the course, and confidence in becoming a real doctor, there is no statistical significance (*P* > 0.05).

**Table 1:** The results of the Competency Improvement Satisfaction Questionnaire [n (%)]

	Study group(*n* = 34)			Control group(*n* = 33)			*P*	*x*^*2*^
	yes	neutral	no	yes	neutral	no		
Improve learning interest	27(79.4)	6(17.6)	1(2.9)	17(51.5)	12(36.4)	4(12.1)	0.048	6.059
Improve independent learning ability	30(88.2)	2(5.9)	2(5.9)	20(60.6)	9(27.3)	4(12.1)	0.029	7.108
Improve teamwork ability	28(82.4)	4(11.8)	2(5.9)	23(69.7)	8(24.2)	2(6.1)	0.405	1.809
Improve clinical thinking ability	25(73.5)	6(17.6)	3(8.8)	18(54.5)	11(33.3)	4(12.1)	0.254	2.739
Improve interpersonal communication ability	28(82.4)	5(14.7)	1(2.9)	18(54.5)	11(33.3)	4(12.1)	0.045	6.210
Improve active problem solving ability	28(82.4)	4(11.8)	2(5.9)	17(51.5)	12(36.4)	4(12.1)	0.025	7.342
Improve active knowledge acquisition ability	26(76.5)	4(11.8)	4(11.8)	19(57.6)	10(30.3)	4(12.1)	0.162	3.646
Improve language expression ability	26(76.5)	6(17.6)	2(5.9)	21(63.6)	9(27.3)	3(9.1)	0.518	1.317
Improve understanding knowledge points in the course	25(73.5)	7(20.6)	2(5.9)	19(57.6)	10(30.3)	4(12.1)	0.368	2.000
Improve confidence to become a real doctor	21(61.8)	9(26.5)	4(11.8)	17(51.5)	10(30.3)	6(18.2)	0.651	0.859

The results of ACIR are shown in Table 2. The study group is significantly better than the control group in organization, timeline, transition statements, open questioning, smooth progress, avoid repetition, summarizing, understandable language, documentation and total score, and the differences are statistically significant (*P* < 0.05). There are no significant difference in the scores of eye contact and no interruption (*P* > 0.05). There are differences between the two groups in terms of response to concerns, positive feedback, and additional questions, but the differences are not statistically significant (P > 0.05).

**Table 2:** The scores of ACIR

	Study group	Control group	*P*	*t*
Organization	3.35 ± 0.69	2.85 ± 0.67	0.003	3.038
Timeline planning	3.15 ± 0.66	2.70 ± 0.73	0.010	2.657
Transition statements	3.91 ± 0.57	3.48 ± 0.62	0.005	2.938
Open questioning	4.18 ± 0.58	3.82 ± 0.53	0.010	2.653
Smooth progress	3.97 ± 0.76	3.45 ± 0.67	0.004	2.957
Avoid repetition	4.21 ± 0.64	3.73 ± 0.72	0.005	2.878
Summarizing	3.32 ± 0.77	2.76 ± 0.75	0.003	3.049
Understandable language	4.41 ± 0.50	3.85 ± 0.71	0.000	3.756
Documentation	2.12 ± 0.81	1.67 ± 0.60	0.012	2.596
Eye contact	4.35 ± 0.49	4.18 ± 0.64	0.219	1.242
No interruption	4.21 ± 0.73	4.00 ± 0.79	0.272	1.108
Response to concerns	1.88 ± 0.54	1.67 ± 0.48	0.088	1.733
Positive feedback	2.35 ± 0.60	2.09 ± 0.58	0.073	1.823
Additional questions	1.91 ± 0.67	1.67 ± 0.48	0.090	1.721
Total score	47.32 ± 2.64	41.91 ± 2.30	0.000	8.952

**Fig. 2:**
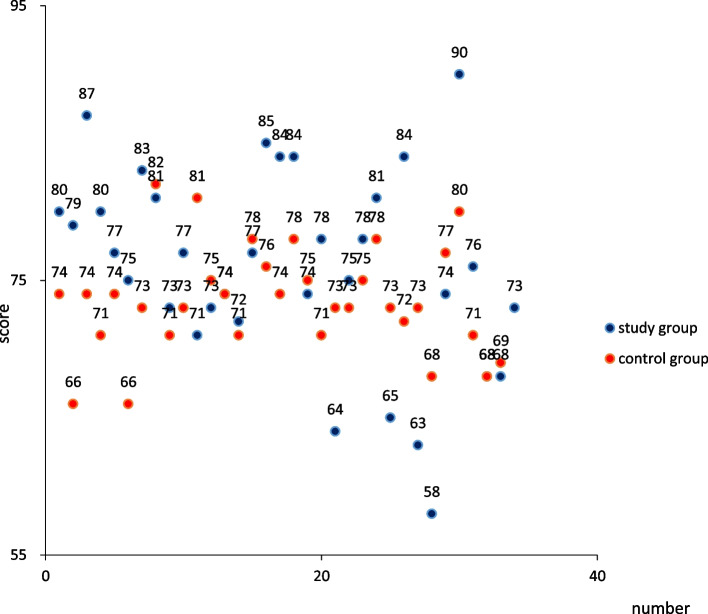
The scores of theoretical test

The theoretical test results after the course are shown in Fig. 2 and Table 3. The results were 76.35 ± 6.83 in the study group and 73.55 ± 3.90 in the control group, using independent sample T test, *P* = 0.043<0.05, the results are statistically different.

**Table 3:** The scores of theoretical test

	Study group	Control group
scores	76.35 ± 6.83	73.55 ± 3.90
*P*	0.043	
*t*	2.058	

## Discussion

The key factor in ensuring and enhancing the quality of health systems is high-level education that transforms learners into qualified health professionals [21]. Traditional training is the common pedagogical method for learning clinical skills, and the aim of medical educators is to identify the best methods for undergraduates to prepare for their professional career [22]. In recent years, many countries promote competence-oriented medical education [23]. The reform of medical education in China also emphasizes the importance of post competency. In this study, we adopted self-feedback (SF), PF and EF modes in the feedback link of the study group and control group. The difference between the two groups was that the study group adopted a new method combining VF with PRP, while the control group adopted a conventional method. At the end of the course a self-developed questionnaire was distributed to investigate students’ satisfaction with this teaching method to improve their ability, and we also used ACIR to assess students’ clinical inquiry ability and communication skills. ACIR was selected because it proved reliable and valid for accuracy both world-wide and domestically and it has also been used continuously until now in many communication studies [24, 25]. Theoretical knowledge is the basis of clinical competency, so we carried out theoretical testing in this study.

Video feedback combined with peer role-playing could avoid the shortcomings of a single teaching method. PF was influenced by prevalent feedback culture, interpersonal consent, and the quality of relationships, and constrained by avoiding criticism and maintaining work relationships at the same time [26], and the quality of feedback may also be questioned by the lack of expertise in peer feedback. The dual identity of peer role player and peer feedback and the feedback from the expert could dispel the doubts about the quality of feedback. VF allows students to directly observe their own performance, objectively assess their learning gap, compare peers’ performance, and avoid problems such as recall bias [11].

Video feedback combined with peer role-playing could stimulate interest in learning actively, and improve interpersonal communication ability. External feedback attracting attention to the task-level has positive effects [27]. In this study, we found that video recording and role play, which are student-centered, make sure students pay more attention on the course. All students make full preparation for it, some find lots of props, some dress in uniform, and others do make-up slightly. The CISQ shows that this novel teaching method combining video recording, PRP, VF and PBL can improve students’ learning interest and autonomous learning ability more than the traditional teaching method. PRP which is proved to achieve similar results as using standardized patients in medical education [28, 29] creates an environment that is similar to the process of clinical inquiry, diagnosis and treatment to combine theory with clinical practice, and the equal status of peers can make learners focus more on the feedback process and content and share and discuss their observations more spontaneously. Learners will spontaneously produce an evaluation and self-regulated learning process from the feedback of teachers, peers, videos, etc., and internal feedback will be generated continuously in this process [30]. The combination of internal and external feedback is more conducive to conceptual learning performance, strategy use, student intrinsic motivation, and perceived competence than providing internal and external feedback alone. VF allow students to directly observe their own and peers’ performance while listening to the content of feedback. VF combined with PRP which promote students to participate in practice and assess their own shortcomings accurately could improve students’ comprehensive analysis ability, clinical performance and communication skills [31, 32], and also can encourage them to participate in and help them to develop their autonomous learning and communicative competence [33].

Video feedback combined with peer role-playing could improve learning efficiency and clinical knowledge and skills. In our study, the questionnaire shows that the study group is better than the control group in terms of improving teamwork ability, cultivating clinical thinking ability, actively acquiring knowledge ability, language expression ability, understanding knowledge points in the course, and is superior to the control group in improving interpersonal communication ability and active problem solving ability. ACIR shows that the study group is significantly better than the control group in organization, timeline, and transition statements, open questioning, smooth progress, and avoid repetition, summarizing, understandable language, and documentation. The learning process can be viewed as a transition from external feedback to internal monitoring, in which individuals are prone to make mistakes. Video feedback combined with peer role-playing teaching method, a transitional learning process from external feedback to internal monitoring, could helps to detect and reduce the errors made by individuals [27, 34]. PRP can simulate the situation of facing real patients, meanwhile, video recording and feedback can review various details and their own performance in PRP from different perspectives, enable students to discover mistakes they did not realize before and conducive to self-reflection [35]. Research has shown that feedback that highlights errors is associated with better skill learning than feedback that shows correct performance [36]. High-information feedback is the most effective because it helps students understand not only the mistakes they have made, but also why they have made and how to avoid making again. Moreover, research shows that teacher-to-student feedback and student-to-student feedback are very effective [37]. A study demonstrates that external feedback results in a higher level of mastery technical skills [38]. Through repeated presentation, it is not inferior to face-to-face guidance from experts in improving clinical knowledge and skills of medical students [39]. The self-analysis, evaluation and feedback of PRP videos and the summary, analysis, evaluation and feedback of instructors can not only improve learning efficiency as effectively as the feedback from experts [40], but also provide additional repeatable learning resources for students [23]. In this way, the clinical experience of medical students can be enriched and their learning ability and proficiency in clinical skills can be improved [41, 42]. Video feedback can record students’ learning behavior and feed back to them, which can slow down the forgetting speed of acquired knowledge and skills. Researches show that [43, 44] video feedback method is of great help to improve the clinical training scores of medical students and their long-term mastery of clinical knowledge. In this research, the study group significantly outperformed the control group at the total score of ACIR and the theoretical test scores at the end of the course.

Some students would have negative emotions during feedback, and some students seek additional feedback from teachers, which can help students understand the feedback more clearly, reduce negative emotions, improve self-confidence, and thus improve performance [45]. External feedback only plays an important role in the early stage of learning, but with the increase of individual knowledge, internal monitoring, which not only reflects the information of external feedback, but also promotes the generation of internal feedback, plays a more critical role in the later stage of learning [46]. Learning based on external and internal feedback operates on a common neural mechanism, the internal feedback signal has been operationalized in the form of a confidence reports, and in the absence of external feedback, and the individual’s sense of pleasure and satisfaction is related to confidence in their own actions [47]. In terms of improving the confidence of becoming a real doctor, the satisfaction of the two groups of students was 70.6 and 51.5%, respectively. The study group is better than the control group, which is lower in the two groups although the ratios are both over 50%. On the one hand, it shows that students are not confident enough to face real patients; on the other hand, the teaching method of video feedback combined with PRP cases is helpful to enhance professional confidence of medical students [48]. This suggests that we should carry out more such exercises on the premise of improving students' feelings of pleasure and satisfaction, based on improving students' self-confidence.

## Limitations of the review

A research shows [49] that it is effective for medical undergraduates to use pre-recorded video and role-play to promote their counseling skills. This study also shows that video feedback combined with PRP is obviously superior to the control group in the teaching mode of PBL for medical students, which is worth promoting. However, the limitations lie on the aspects following stated. Initially, the sample size is insufficient and the grouping is not meticulous enough. Secondly, the questionnaire is prepared by the authors according to the teaching practice and students’ feedback, rather than standardized questionnaires. Last but not least, only ACIR was performed to assess clinical communication skills. In short, further research is anticipated.

## Conclusions

It is beneficial to use multiple feedback methods in the teaching process, and the shortcomings of a single teaching method can be avoided and the effectiveness of teaching tools can be improved by combining VF and PRP. At the same time, the combination of VF and PRP is also conducive to the use of the advantages of external feedback and internal feedback and the combination of internal feedback and external feedback, so as to play a positive role. VF combined with PRP teaching method in medical undergraduates adopting PBL teaching mode in China is effective, it can stimulate interest in learning actively, improve interpersonal communication ability, improve learning efficiency and clinical knowledge and skills, and improve the confidence of becoming a real doctor.

## Data Availability

The datasets used and analyzed during the current study are available from the corresponding author on reasonable request.
